# Metastatic gastric squamous cell carcinoma in a western grey kangaroo

**DOI:** 10.1177/10406387251384945

**Published:** 2025-11-03

**Authors:** Andreu Masdefiol Garriga, Barbara Ferreira, Simon J. Girling, Stephanie M. Mota, Linda R. Morrison

**Affiliations:** Easter Bush Pathology, Royal (Dick) School of Veterinary Studies and the Roslin Institute, Easter Bush Campus, nr Roslin, Midlothian, UK; Royal Zoological Society of Scotland, Edinburgh, Scotland, UK; Royal Zoological Society of Scotland, Edinburgh, Scotland, UK; Royal Zoological Society of Scotland, Edinburgh, Scotland, UK; Easter Bush Pathology, Royal (Dick) School of Veterinary Studies and the Roslin Institute, Easter Bush Campus, nr Roslin, Midlothian, UK

**Keywords:** kangaroos, neoplasia, squamous cell carcinoma

## Abstract

An adult male western grey kangaroo (*Macropus fuliginosus*) developed lameness, a stiff gait, and weight loss, and deteriorated despite medical treatment. Postmortem examination revealed a primary gastric squamous cell carcinoma (SCC) with associated cardiac, pulmonary, diaphragmatic, hepatic, and vertebral metastases with lytic bone lesions. Before histologic examination, the macroscopic appearance of the liver lesions had raised concerns about mycobacteriosis. Metastatic gastric SCC has not been reported previously in a western grey kangaroo, to our knowledge

An 8.5-y-old male western grey kangaroo (*Macropus fuliginosus*) had been bred in captivity and was moved to the Edinburgh Zoo (Scotland, UK) in December 2016. The individual was housed with 5 other kangaroos and 9 swamp wallabies (*Wallabia bicolor*) in a mixed indoor-outdoor enclosure. The diet consisted of grass from the enclosure, macropod pellets (Vitality; Waterhouse), DK zoological leaf eater biscuit (Kiezebrink) once a day, as well as daily servings of carrots, cabbage, spring greens, and grass hay. Yellow salt licks (Rockies) were available ad libitum.

In late December 2023, the kangaroo had an abnormally stiff and slow gait, poor coat quality, and loss of body condition. Despite medical intervention, including anti-inflammatory treatment, the animal’s condition progressively worsened, and the lameness remained refractory to treatment, which consisted initially of meloxicam (Inflacam equine oral suspension, 0.6 mL, 15 mg/mL, PO, q24h; Virbac), later combined with tramadol (Zydol, 100 mg, q12h; Grünenthal). A fecal analysis on 2024 January 9 revealed a high (7,150 eggs/g of feces) burden of strongyle eggs, which was treated with an anthelmintic (Panomec, ivermectin 1%; Boehringer Ingelheim). On January 17, the animal underwent general anesthesia for further investigation of the weight loss and lameness. It was darted IM on the left hindlimb with ketamine (Ketamidor, 2 mL, 100 mg/mL; Richter Pharma), medetomidine (Medetomidine HCL, 0.1 mL, 20 mg/mL; Bova), and midazolam (0.5 mL, 5 mg/mL; Hameln Pharma).

Radiographs of the hindlimbs, spine, and thorax revealed a loss of opacity in the right transverse process of T4 (Fig. 1). Additionally, an area of increased opacity was observed at the base of the heart. Examination of the oral cavity revealed severe periodontal disease with moderate gingivostomatitis, which required extraction of several incisors. A blood examination revealed moderate anemia (PCV 0.35 L/L, RI: 0.44–0.51 L/L; RBC 3.8 × 10^12^/L, RI: 4.8–5.7 × 10^12^/L),^1^ moderate hypercalcemia (3.86 mmol/L; RI: 2.17–2.22 mmol/L),^20^ and mild hypophosphatemia (1.55 mmol/L; RI: 1.77–2.15 mmol/L).^20^ Progressive worsening of the gait and overall clinical signs, coupled with a poor prognosis and compromised welfare, led to the decision for euthanasia, which was carried out on January 19 with intravenous pentobarbital (Pentoject 20%, 80 mL; Animalcare) following darting with the same analgesic combination described above.

**Figure 1.**
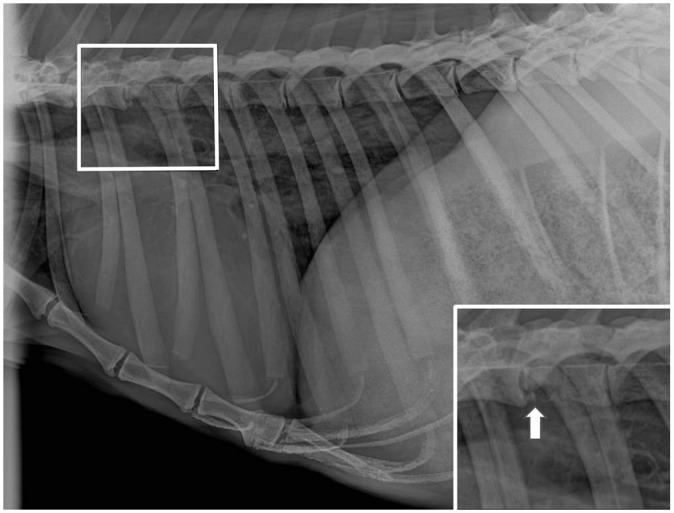
Lateral radiograph of the spine, thorax, and cranial abdomen of a Western grey kangaroo, with focal loss of opacity in T4 (in box). Inset: lytic T4 vertebral lesion highlighted (arrow).

Postmortem examination was performed on the same day. The animal weighed 61 kg and was in very lean body condition. Significant postmortem findings included severe chronic dental disease and chronic degenerative joint disease, with cartilage thinning and degeneration in the elbow joints, left hip, and right stifle. There was moderate serosanguineous abdominal effusion with regionally extensive fibrinous peritonitis resulting in adhesions between a 5-cm ulcerated area of gastric mural thickening and the left liver lobe. The ulceration and mural thickening involved the junction of the nonglandular and glandular gastric mucosa (Fig. 2A). The liver also had coalescing, variably sized (0.1 to ~10-cm), pale-yellow, firm-to-granular and gritty nodules throughout all lobes (Fig. 2B). The macroscopic appearance of the liver lesions resembled the mineralized caseous lesions of mycobacteriosis in marsupials, which may be caused by many different mycobacterial species.^2,6,13^ In impression smears of the liver lesions stained with Ziehl–Neelsen, large numbers of squames and necrotic debris were found, but no acid-fast organisms were identified. The abdominal surface of the diaphragm had numerous, firm, off-white to faint-yellow, 0.2–1.5-cm nodules, with similar 0.2–0.4-cm nodules throughout the lung lobes. A moderate, serosanguineous pericardial effusion was present. The epicardium of the left ventricle had a focal irregularly nodular area, mottled white-to-tan, which was slightly gritty (Fig. 2C). On sectioning, this discoloration extended into the myocardium (~1.5 × 1 cm).

**Figure 2.**
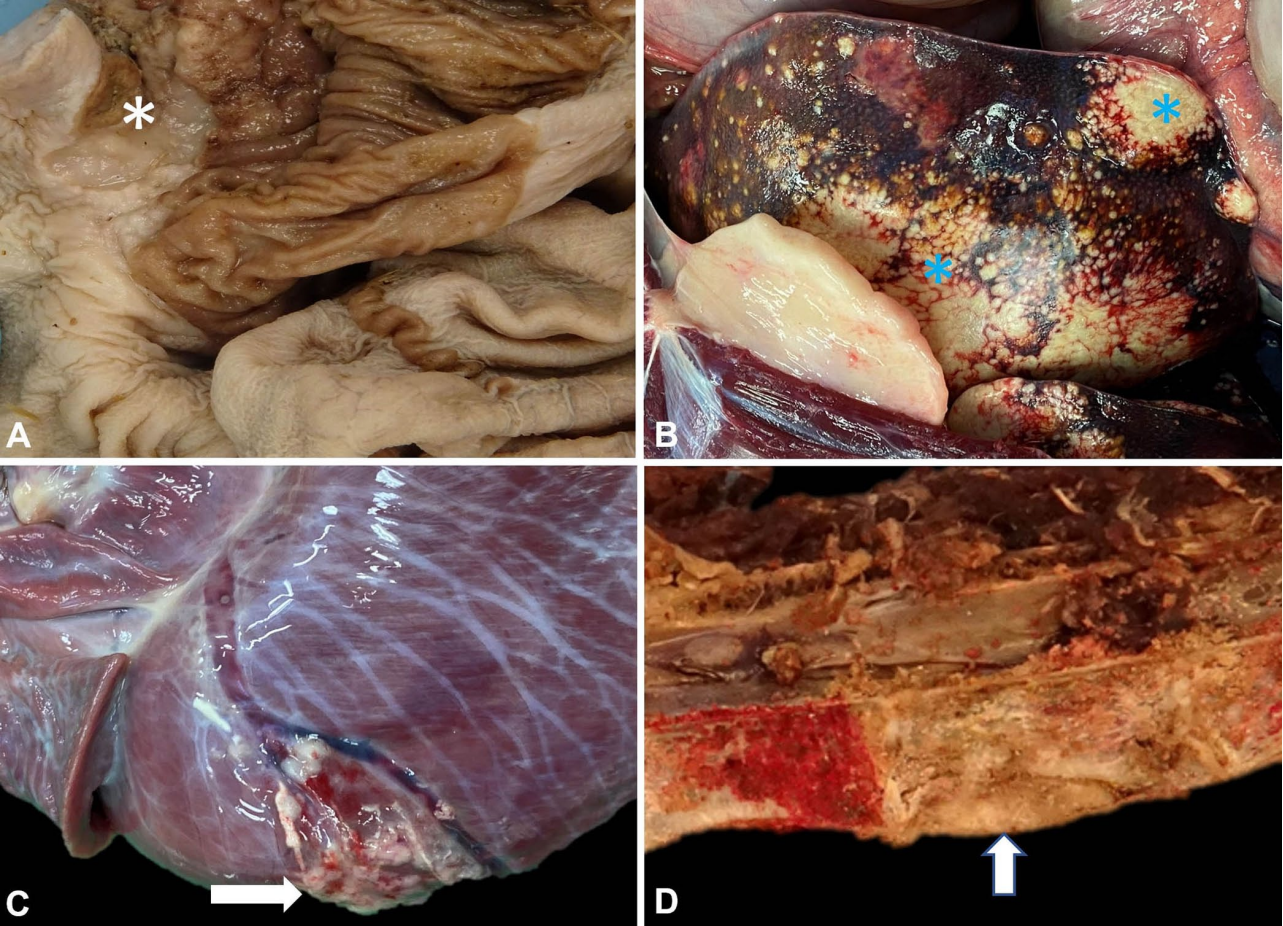
Gastric squamous cell carcinoma in a Western grey kangaroo. **A.** Formalin-fixed stomach, with mucosal ulceration (asterisk). **B.** Coalescing hepatic nodules (asterisks), which were gritty on sectioning. **C.** Focal irregular nodular area (arrow) on the surface of the left ventricle. **D.** Thoracic vertebrae T3, T4, and T5, with a well-demarcated area of softening on the ventral aspect of T4 (arrow).

The vertebral column had a slightly softer area on the ventral aspect of T4 (Fig. 2D). Following initial fixation in 10% neutral-buffered formalin and sectioning, the vertebral bone was mottled white-to-tan in this area. After initial fixation, the vertebral bone sections required demineralization (Surgipath decalcifier I; Leica) before processing. Samples of heart (left and right ventricle), lung, diaphragm, liver, spleen, esophagus, stomach, small intestine, large intestine, thyroid gland, adrenal gland, kidney, urinary bladder, and thoracic vertebra were collected into 10% neutral-buffered formalin, processed routinely, and sections stained with H&E.

Sections taken from the ulcerated and thickened area of the stomach contained an unencapsulated and infiltrative proliferation of atypical cells, effacing and replacing pre-existing tissue and extending transmurally (Fig. 3A–C). Cells were arranged in nests and islands, composed of cuboidal-to-polygonal cells with a variable amount of amphophilic, finely granular cytoplasm. There were multifocal areas of haphazard squamous differentiation, infrequent keratin pearl formation, and frequent necrosis, consistent with a diagnosis of a gastric squamous cell carcinoma (SCC). Nuclear chromatin was clumped-to-vesicular with large nucleoli, and up to 7 mitotic figures were noted in 2.37 mm^2^ (Fig. 2C). There was associated stromal fibrosis (desmoplasia) and mild-to-moderate chronic inflammation, which extended into the attached mesentery. There was also marked, regionally extensive expansion of the serosa by granulation tissue with associated florid fibrinonecrotic debris and neutrophilic inflammation.

**Figure 3.**
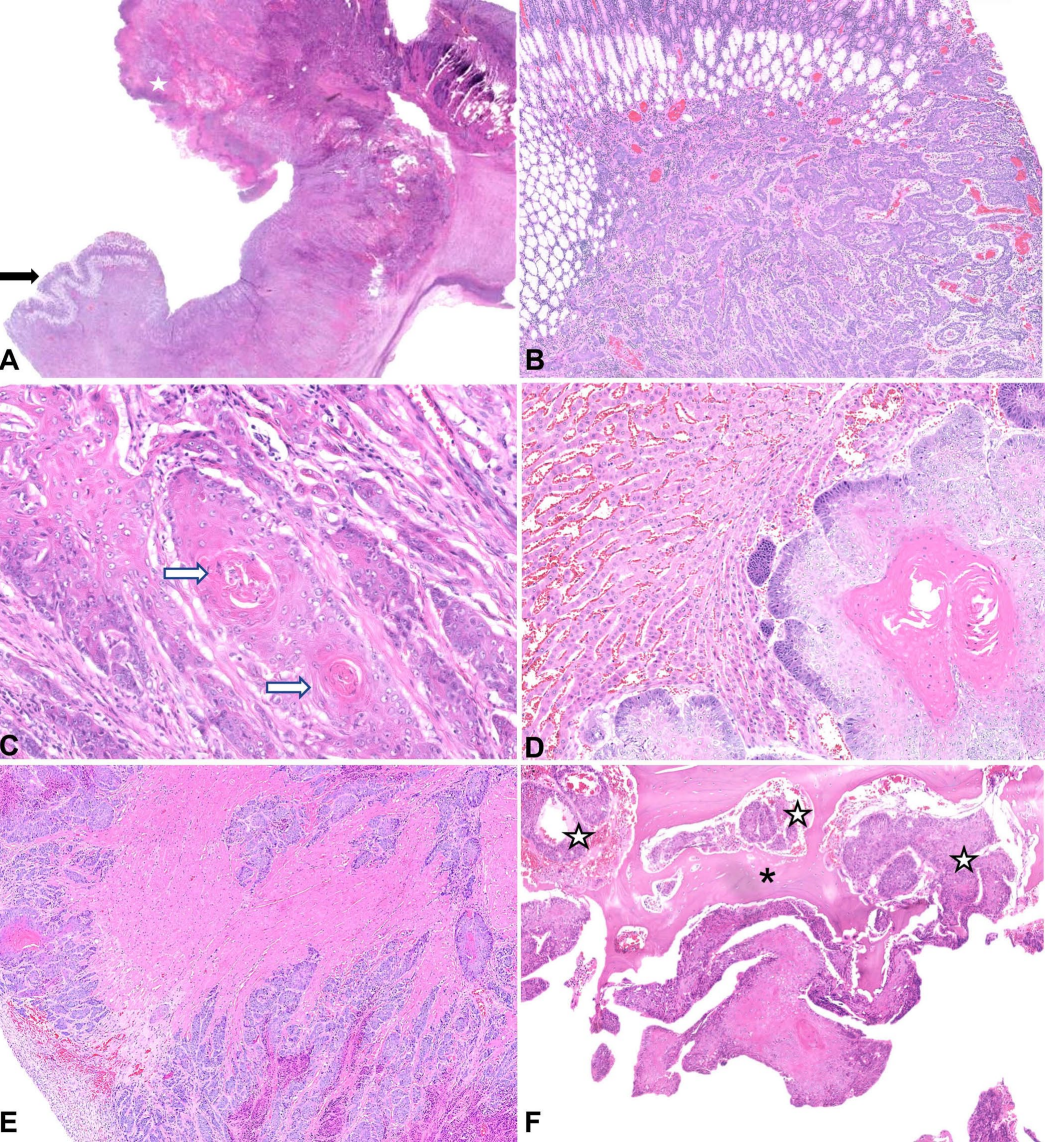
Microscopic images of gastric squamous cell carcinoma (SCC) in a western grey kangaroo. H&E. **A.** Subgross image of the stomach; a small area of normal gastric mucosa (arrow) with replacement of the rest of the tissue by the neoplastic proliferation. **B.** Nests of pleomorphic epithelial cells infiltrating the gastric mucosa. **C.** Within the nests of pleomorphic epithelial cells, there is squamous differentiation and infrequent keratin pearls (arrows). **D.** Metastases of the gastric SCC in the liver. **E.** Coalescing myocardial metastases. **F.** Tumor invasion (white stars) into thoracic vertebra T4 (asterisk).

The lung, liver, diaphragm, myocardium, and vertebral body of T4 also contained coalescing nests and islands of the atypical cells described above with haphazard squamous differentiation, keratinization, and necrosis (Fig. 3D–F). In addition, within the myocardium, there was mild, multifocal interstitial fibrosis and chronic (mainly lymphocytic) inflammation. On the capsular surface of the spleen and visceral surface of the diaphragm, there were reactive mesothelial cells, consistent with peritonitis. Mild, chronic interstitial nephritis with periglomerular fibrosis and tubular mineralization, and eosinophilic enterocolitis were also noted.

Neoplasia is reported infrequently in kangaroos, with oral SCC and mammary adenocarcinomas described as the most frequent neoplasms.^16^ To our knowledge, disseminated SCC of gastric origin has not been described previously in a kangaroo; we retrieved no cases of metastatic gastric SCC in a search of PubMed, Google Scholar, CAB Direct, Scopus, and Web of Science, using the search terms “gastric squamous cell carcinoma” and “kangaroo”, suggesting that this condition has not been reported previously in western grey kangaroo. Our case had a primary gastric mass, histologically confirmed as a gastric SCC with widespread metastases in the lung, liver, diaphragm, myocardium, and thoracic vertebral column. As a result of the underlying neoplasia, the animal was in extremely poor body condition and had pericardial and abdominal effusions. The vertebral metastasis likely contributed to the observed slow, stiff gait and lameness; the reduction in skeletal musculature can be attributed to cancer cachexia.

A case of SCC in the gastric fundus of a red kangaroo (*Macropus rufus*) is mentioned in the literature, although with no description provided.^7^ Gastric SCC has also been described in a pademelon (*Thylogale billardierii*).^10^ In the pademelon, there was a papillomatous outgrowth in the mucosa of the stomach with numerous intralesional nematode larvae, identified as *Labiostrongylus* sp., but no metastases were found. However, no histologic analysis of tissues other than the stomach was performed.^10^ Other locations in which SCCs have been identified among members of the *Macropodidae* family are the cervix and vagina,^5^ oral cavity,^14^ and base of the pinna.^3^ SCCs found in individuals in the *Macropodidae* family, mainly in the oral cavity, are described as locally malignant tumors, with destruction of bone and invasion of adjacent oral tissues, although metastases are also reported (e.g., a 10-y-old female red kangaroo was diagnosed with gingival SCC with metastasis in the mandibular lymph node^14^). Another case of an adult red kangaroo diagnosed with oral SCC revealed extensive areas of bone destruction in the maxillary and palatine bones.^14^

The stomach of western gray kangaroos is comprised of a sacciform forestomach, a tubiform forestomach, including a non-glandular and a glandular part, and a hind stomach, where the fundus and pylorus are located.^15^ In our case, it is likely that the tumor originated from the nonglandular part of the tubiform forestomach and extended to involve the glandular portion. In the horse, which is the domestic species that is most commonly affected by this type of neoplasm in the stomach,^11,17^ SCCs are predominantly described as arising in the nonglandular mucosa.^9,15,18,19^ SCCs are considered common tumors in domestic animals, such as dogs, cats, horses, and cattle, and most frequently develop in the skin, oral cavity, esophagus, and mucocutaneous junctions.^4,11,12^ In one study, 68% of horses with gastric SCC had metastases, and clinical signs were often nonspecific, but included weight loss and lethargy, as seen in our case.^17^

Hypercalcemia may be due to the humoral hypercalcemia of malignancy and has been reported in association with several malignancies in animals, including gastric SCC in horses.^11,14,17^ Other possible causes of hypercalcemia include metastasis of solid tumors to bone, granulomatous inflammation, renal disease, vitamin D toxicosis, and primary hyperparathyroidism.^11^

As marsupials are reported to be susceptible to mycobacteriosis,^2,6,8^ and mycobacterial osteomyelitis has been recognized in several species, the presence of caseous lesions in the liver, along with the clinical history of hypercalcemia, led to the consideration of mycobacteriosis in this animal at the time of postmortem examination. No evidence of granulomatous inflammation or acid-fast organisms was observed in impression smears of the liver, and the histologic findings of the lesions confirmed metastasis of the gastric SCC.
